# The niche of One Health approaches in Lassa fever surveillance and control

**DOI:** 10.1186/s12941-021-00431-0

**Published:** 2021-04-24

**Authors:** Liã Bárbara Arruda, Najmul Haider, Ayodeji Olayemi, David Simons, Deborah Ehichioya, Adesola Yinka-Ogunleye, Rashid Ansumana, Margaret J. Thomason, Danny Asogun, Chikwe Ihekweazu, Elisabeth Fichet-Calvet, Richard A. Kock

**Affiliations:** 1 Centre for Clinical Microbiology, Division of Infection and Immunity, University College London, London, UK; 2 The Royal Veterinary College, University of London, Hatfield, UK; 3 Natural History Museum, Obafemi Awolowo University, Ile Ife, Nigeria; 4 Institute of Lassa Fever Research and Control, Irrua Specialist Teaching Hospital, Irrua, Nigeria; 5 Department of Microbiology, Ambrose Alli University, Ekpoma, Nigeria; 6 Nigeria Centre for Disease Control, Abuja, Nigeria; 7 School of Community Health Sciences, Njala University, Bo, Sierra Leone; 8 Department of Virology, Bernhard Nocht Institute for Tropical Medicine, Hamburg, Germany

**Keywords:** Lassa fever, One Health, Zoonosis, Emerging infectious diseases

## Abstract

Lassa fever (LF), a zoonotic illness, represents a public health burden in West African countries where the Lassa virus (LASV) circulates among rodents. Human exposure hinges significantly on LASV ecology, which is in turn shaped by various parameters such as weather seasonality and even virus and rodent-host genetics. Furthermore, human behaviour, despite playing a key role in the zoonotic nature of the disease, critically affects either the spread or control of human-to-human transmission. Previous estimations on LF burden date from the 80s and it is unclear how the population expansion and the improvement on diagnostics and surveillance methods have affected such predictions. Although recent data have contributed to the awareness of epidemics, the real impact of LF in West African communities will only be possible with the intensification of interdisciplinary efforts in research and public health approaches. This review discusses the causes and consequences of LF from a One Health perspective, and how the application of this concept can improve the surveillance and control of this disease in West Africa.

## Background

The Coronavirus Infectious Disease 2019 (COVID-19) emergence since December 2019, and the subsequent pandemic, has highlighted that over recent decades changes in human and domestic animal demographics and their associated impacts on the environment have fundamentally affected ecosystem dynamics [1]. The One Health concept and its 12 “Manhattan Principles” [2], elaborated in the 2000s, have been warning stakeholders how anthropic actions affect global health and the urgency of measures to prevent Emerging Infectious Diseases (EIDs) and, ultimately, a pandemic. Pathogenic viruses, bacteria and fungi continue to evolve under changing natural selection pressures and have become an existential threat, with a huge socio-economic burden, such as the HIV and COVID-19 pandemics. Moreover, humanity is still struggling with endemic high-consequence pathogens (e.g. Plague, Hantaviruses), which seem invisible for most of the world and are increasingly neglected in the face of novel pandemics.

There is an epidemiological link to domestic animals and/or captive wildlife in food systems, farms or trade, which incubate or vector evolving pathogens and bring them into close contact with humans. Free-living animals (wildlife) are not typically a direct zoonotic source of human disease, except for rare cases when the wildlife has adopted a peri-domestic behaviour, exploiting food and habitat created by humans [3]. For instance, Lassa fever (LF) is a viral haemorrhagic infection for which the main reservoir is the Natal multimammate mouse (*Mastomys natalensis*), a now semi-domesticated African rodent associated with human settlement in forested environments [4–6].

The infectious agent of LF is the *Lassa mammarenavirus* (LASV), an enveloped single-stranded RNA member of the *Arenaviridae* family [7]. Its genome is segmented into Small (3.4 Kb) and Large (7 Kb) RNA fragments. The L RNA segment encodes the viral polymerase (L protein) and a zinc-binding protein (Z). The S segment encodes the nucleoprotein (NP), and the glycoprotein precursor (GPC) which will be cleaved into the membrane proteins GP1 and GP2 [8]. Both NP and GP1/GP2 proteins are the main targets for diagnostics and phylogenetics approaches.

This disease is endemic to West Africa with outbreaks reported from Nigeria, Sierra Leone, Liberia, Benin and Togo in recent years [9]. Nigeria, with annual outbreaks, continues to report the greatest number of cases in the region with peak incidence of LF cases reported in January to March [10]. However, the increased numbers may in part be due to the dramatic improvement in testing capacity under a dedicated program by the Nigerian Centre for Disease Control (NCDC) [11].

Zoonotic transmission of LF caused by spillover from the reservoir species is the primary driver of human cases of the disease, however, human-to-human transmission chains have been described [12–14]. These chains, while relatively uncommon, are typically associated with nosocomial settings and many of these were related to a small number of ‘super-spreading’ events. Hence, the zoonotic origin of LF and its transmission requires a One Health approach with sustained commitment to its control. This review provides a One Health perspective of LF, outlining how human health, peri-domestic animal ecology (facilitated by the virus and rodent-host genetics) and environmental factors affect outbreaks of this disease in West Africa.

## Geographical distribution of Lassa fever

Lassa fever is endemic in several countries of West Africa, primarily the Mano River Region states of Sierra Leone, Guinea, and Liberia [15]. However, Nigeria continues to suffer the greatest burden of disease in terms of number of reported cases [16, 17]. Sporadic human cases of disease have been also reported from Togo, Benin Republic, Cote d’Ivoire, and Mali [18, 19].

Molecular epidemiology has so far revealed seven distinct lineages of the LASV, including four confirmed and three proposed lineages [20–24] suggesting localisation of infection around specific human-rodent communities and socioecology. Lineages I, II and III are found in Nigeria [22]. Lineage I was described from the surrounding areas of Lassa village in north-eastern Nigeria, where the first LF case was reported in 1969 but has not since been detected. Lineages II and III are common in southern and north-central Nigeria respectively [22]. Lineage IV, which includes the Josiah strain, comprises the subclades IVa and IVb, predominant in the Mano River Union area (Sierra Leone, Liberia and Guinea) [20]. In Sierra Leone, LF is endemic in the Eastern Province, particularly Kenema District [24], but has been reported in almost all districts of the country. In Liberia, LF is predominant in the Lofa, Grand Cape Mount and Nimba Counties [13], but several other counties have had cases of LF in the recent past [13, 24, 25]. Lineage V is a proposed lineage for LASV strains from Côte d'Ivoire and Mali. The sixth lineage, associated with Nigeria, comprises the “Kako” strains, while the seventh proposed lineage is linked to Togo [20].

Increasing numbers of reported cases in the West African endemic region has raised concerns over the export of LF to countries outside the region [7]. From the first case in 1969 until 2020, at least 35 exported cases of LF have been reported. These cases were exported from seven West African countries to nine countries in Europe, Asia, South Africa and North America [21, 26] and several individuals have been repatriated due to the risk of LF transmission during outbreaks in endemic areas, or exposure during medical procedures [27, 28].

It is noteworthy that LF cases exported from West Africa have been reported in various parts of the world. However, no local transmission has been recorded in the countries where the cases were identified [7, 19, 29]. Recent modelling work suggests that the countries outside of West Africa at greatest risk of importation of an individual with LF are the United States of America, United Kingdom, United Arab Emirates and South Africa. In models of the risk of disease export, the majority (61%) of simulated outbreaks had no exported cases, with a single imported case in 30% of simulations [30]. These simulations are sensitive to the total number of annual cases and the peak number of cases during seasonal outbreaks. This work did not incorporate the age or socio-economic position of individuals infected with LF in risk calculation.

## Rodent dynamics and Lassa fever outbreaks

**Fig. 1:**
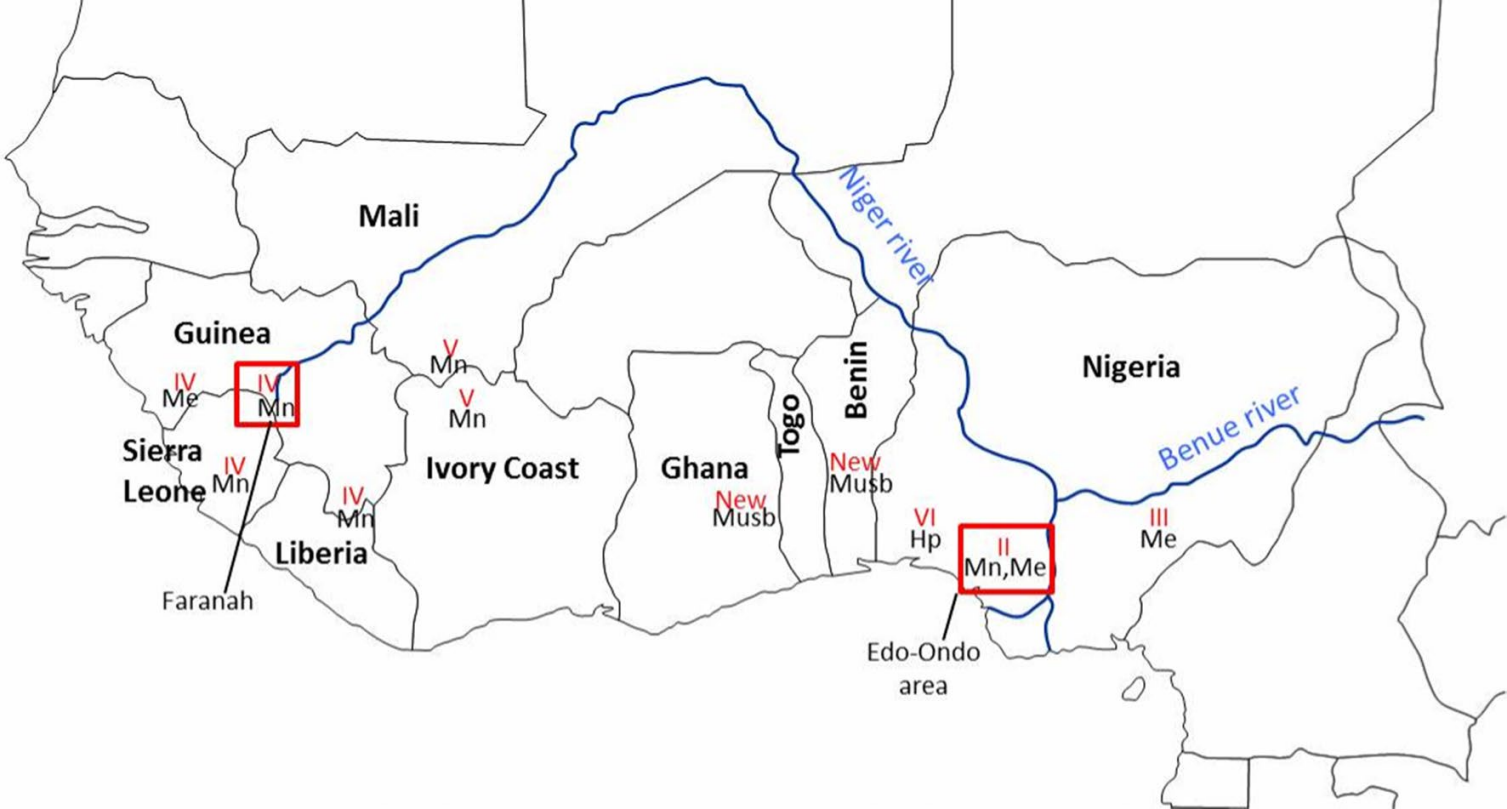
LASV lineage distribution across West Africa in relation to rodent host species. Virus lineages appear in red. Rodent taxa are abbreviated as *Mn* *Mastomys natalensis*, *Me Mastomys erythroleucus*, *Hp* *Hylomyscus pamfi*; *Musb* *Mus baoulei*. Brackets toward the bottom describe the geographical ranges of *Mastomys natalensis* mitochondrial lineage A-I (across West Africa) and A-II (extending into Central Africa). Red boxes indicate examples of LASV hotspots mentioned in this review where fine-scaled genetic studies have already begun

Delineating LASV occurrence and lineage assortment in rodent populations across West Africa is important for anticipating emergence at the regional level, but determining how strains of this virus circulate among several sites and localities within a specific hotspot can help chart transmission during outbreaks. One of the early studies on LASV microevolution in its natural reservoir [31] analysed 132 partial nucleoprotein sequences of LASV from *M. natalensis* trapped in 12 villages in the Faranah area Upper Guinea, over 12 years (Fig. 1). The investigators found that viruses circulating in a specific locality were diverse and polyphyletic to viruses from neighbouring villages. Yet there were monophyletic clusters formed by viruses from a village at specific points in time, indicating that the temporal and spatial pattern of LASV evolution in the natural reservoir was characterised by a combination of stationary circulation within a village and virus movement between villages [31]. Extending this study in Faranah, it was observed that the majority of infected *M. natalensis* were trapped in only a few houses, and the LASV sequences from individuals within the same house were genetically more similar than LASV sequences from rodents captured in different houses of the same village. This suggests a predominance of human-to-human transmission in this particular area, rather than multiple rodent-to-human exposures [32].

Although whole viral genome sequencing is already a common practice for LASV diagnosed in humans [16, 33, 34] the approach also needs to be adopted as a feature to enhance phylogenetic resolution in rodent studies. Indeed, an overriding aspiration in generating LASV sequences from rodents is to be able to link them to those from humans. In an early attempt at direct comparison, Olayemi et al. found intriguingly that human-derived LASV sequences from two highly endemic localities (Ekpoma in Nigeria and Kenema in Sierra Leone) were more ancient phylogenetically in relation to those detected in rodents from the same respective towns, indicating the possibility of reverse zoonosis in LF. The authors suggested that future studies in places like Ekpoma (which is situated within the Edo-Ondo hotspot of Nigeria, Fig. 1) should aim to link human and rodent data more directly at a finer scale. They also recommended that longitudinal sampling regimes be supported by opportunistic rodent screening at home addresses where humans test LASV-positive [35].

Furthermore, indications of the seasonality of LF outbreaks were documented early on in the description of this disease [36]. Increased recognition of LASV cases and improved local testing capacity since the early 2000s has provided further understanding of a seasonal link to zoonotic spillover events and outbreaks [37, 38]. Data from Guinea showed that in the dry season, when the food supply is restricted, *M. natalesis* tend to aggregate within houses. However, the number of LASV-positive rodents was two to three-fold higher in the rainy season than in the dry season. Although transmission from rodents to humans occurs throughout the year, the increased risk of exposure to rodents’ contaminated excreta in the dry season potentially drives the higher rate of human LF cases [12, 39]. A similar effect has been observed with data from Irrua Specialist Teaching Hospital in Nigeria, where the number of LF cases and admissions were consistently higher in the dry season (January-March) throughout seven years [37]. Interestingly, in Sierra Leone, two peaks of LF cases have been reported in both dry (January–March) and rainy (June–August) seasons [24, 40]. Although recent Ebola epidemics, civil conflicts and poor house hygiene have been implicated as driving increased rates of LF cases [24], early observations also showed the lack of correlation of LF outbreaks and dry season in Sierra Leone [40].

The primary driver for LF seasonal pattern is proposed to be the rodent host. Reproduction of the multimammate mouse occurs throughout the year with higher levels of fecundity closely tied to rainfall. Vertical transmission of LF is predicted to occur as LASV can be found in animals of all ages, although horizontal transfer is likely the predominant route of infection [41]. A model of rodent reproduction and population abundance suggests peak population levels being reached between April and May [42], which would correlate with a peak prevalence of LASV in the rodents between March and April.

The precise location of the rodent host within the ecosystem has been observed to vary during the year dependent on the availability of food, with a general migration from agricultural settings to areas of human habitation during the dry season [43]. In Guinea, it has been shown that *M. natalensis* abundance increased within human habitations during the dry season [39]. In Nigeria, on the other hand, *Mastomys spp.* were captured mostly indoors throughout the year, with population peaks occurring for *M. natalensis* at the height of the rains and for *M. erythroleucus* during the dry period [44]. Further, changes in human activity and behaviour may be simultaneously contributing to an increased incidence of LF [45]. Activities such as farming and forestry during the dry season may be exposing individuals to a greater level of risk of viral infection during these periods [46]. Additionally, activities that increase risk such as trapping and hunting rodents may also have seasonal variation that contribute to these observed patterns.

Therefore, understanding of seasonal dynamics of LF may lead to greater rates of testing or increased clinical vigilance of the presentation of this disease in areas where it is known to be endemic [47]. For example, testing capacity and clinician education have been dramatically increased by the NCDC. This expansion in testing capacity in Nigeria likely explains the year-on-year increase in reported LF cases [11]. Despite this, the seasonal differences in cases remain, suggesting a true phenomenon that requires further investigation.

The effect of future changes on the frequency of outbreaks and the impact of climate and land-use change on seasonality is even less clear. Human populations in West Africa continue to grow at an annual rate of 2.7% [48] and despite increasing urbanisation [49], large numbers of the population will remain at risk of LF outbreaks. Increasing agricultural intensification and use of monoculture practices may lead to an increased abundance of generalist rodent pest species such as the reservoir species of LASV [50]. Livestock production and domestic animals such as dogs and cats may have a role in driving outbreaks if they can act as hosts for LASV. Currently, no systematic testing of domestic animals has been performed. Climate and land-use change in the West African region are modelled to lead to intensification and a northward shift of the monsoon system [51] which could lead to a greater abundance of generalist rodent species. Together, these factors could continue to drive and amplify the observed seasonal outbreaks of LF in the region.

### Lassa mammarenavirus reservoirs

**Fig. 2:**
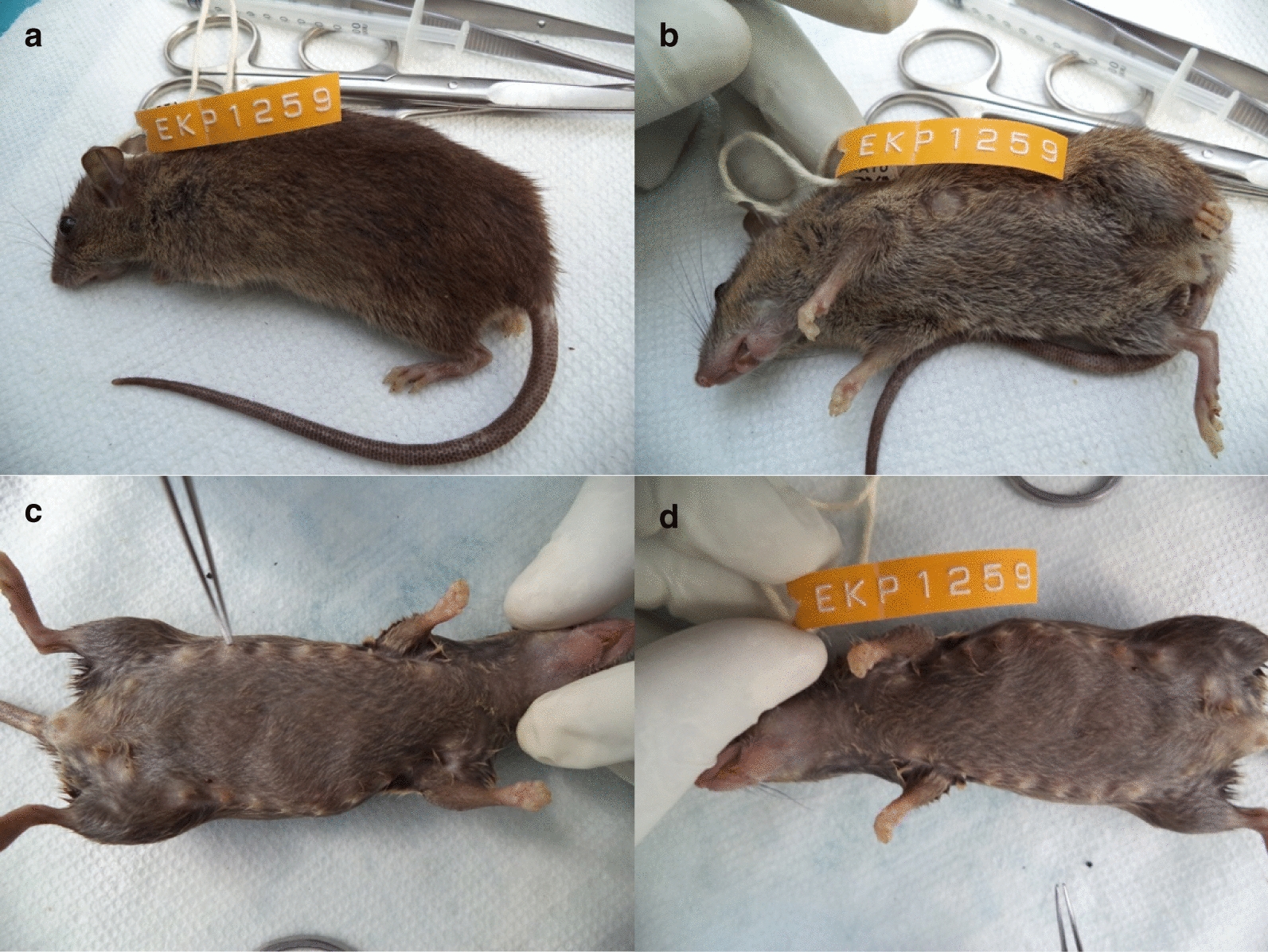
Different aspects of a Mastomys natalensis rodent (**a**–**d**). Speciments were captured during a LASV ecological survey in Nigeria, 2015. Images **c** and **d** show the distinctive high number of nipples (8–12 pairs) present in mature, lactating females. Photo credits: Olayemi et al., Nigeria, 2015

LASV was isolated from peri-domestic rodents for the first time in 1974, during an LF outbreak in Sierra Leone. The virus was isolated from the Natal multimammate mouse (*Mastomys natalensis*) trapped in residents’ houses and surrounding gardens and fields [52]. LASV rodent-to-human transmission occurs directly when humans are exposed to rodent fluids or indirectly through exposure to foodstuff and surfaces contaminated with rodent excreta [53]. Rodent urine has been suggested to be a major risk for human infections [54]. Although *M. natalensis* was first described in South Africa, it is now known to be the most widespread rodent species across sub-Saharan Africa [55–58]. They are rodents with usually 8 to 12 pairs of nipples (Fig. 2); hence the common name ‘multimammate’ which means many-breasted mouse [58]. *Mastomys spp*. occur in many types of habitat*.* They breed frequently, produce large numbers of offspring, and are numerous in open grassland, mixed savannah and clearings in forests. They are also highly adaptable and can be expected to display variation in their ecology and behaviour, thriving in agricultural fields and human dwellings [59, 60]. Due to their behavioural plasticity, *Mastomys* are also considered commensals and are often captured indoors [60].

LASV has also been detected in the Guinea multimammate mouse (*Mastomys erythroleucus*) in certain localities within Nigeria and Guinea, the African wood mouse (*Hylomyscus pamfi*) in Nigeria, and the Pygmy mouse (*Mus baoulei*) in Ghana and Benin, suggesting these species as viable hosts of the virus [61–63]. Other species may have been erroneously named as reservoirs as a result of uncertain taxonomy [64]. There are also small mammals that, at some point, showed seropositivity but have consistently tested negative for active LASV infection in multiple investigations over the years. It is believed that these taxa, such as Dalton’s mouse (*Praomys daltoni*) and Olivier’s shrew (*Crocidura olivieri*), are involved in incidental, spill-over infections because of their proximity to LASV-positive *M. natalensis* [65, 66].

A One Health approach to understanding the ecology of LF will lead to an improved ability to assess the zoonotic risk to humans. Fundamental to this ecology is an accurate depiction of the occurrence of various LASV lineages and strains circulating among reliably identified rodent species and subtaxa. Currently, molecular analysis has provided a broad regional picture of which LASV lineages are distributed across key rodent populations within West Africa (Fig. 1) [67]. This has helped map out hotspots of rodent-borne circulation with the potential for emergence.

*M. natalensis* is distributed all over SSA. Across West Africa, it co-occurs with *M. erythroleucus*. Questions remain concerning why the virus is endemic only in certain populations of these *Mastomys* rodents within West Africa. Mitochondrial Cytochrome *b* DNA is frequently used for species and lineage level designations among murid rodents across Africa and it has also been the marker of choice for the molecular taxonomy of small mammals in many of the LASV ecology studies [68]. Although LASV has not yet been detected in *M. natalensis* in Central Africa, LASV-positive individuals detected within Nigeria indicate that mitochondrial lineage A-II of this rodent, which extends from Nigeria into Central Africa (Fig. 1), is not immune to infection [69]. Mitochondrial lineages, nevertheless, have not been able to explain the patchy occurrence of LASV in *Mastomys* populations across West Africa.

Lalis et al. (2012) used nine unlinked microsatellite loci to assess *M. natalensis* population structure in Guinea. They demonstrated that populations of this rodent in the Faranah and Denguedou areas belonged to a distinct clade compared to those from other parts of the country. Within this clade, however, they were unable to provide genetic evidence that differentiated LASV-positive rodents from the LASV-negative ones [70]. Future studies, combining neutral microsatellites with immunogenetic markers such as those of the Major Histocompatibility Complex (MHC), could provide added insight regarding the role of adaptive genetic diversity in the susceptibility of rodents to LASV infection [71]. Such investigations would illustrate the potential (beyond factors like kinship and geographic proximity to other virus-positive rodents) that a *Mastomys* individual constitutes a competent LASV host. MHC genes code for cell surface glycoproteins that recognise, bind to, and present foreign antigens to T cells, initiating the appropriate immune response. MHC is the most variable part of the genome among vertebrates. It is believed this diversity is important for hosts to recognise antigens presented by a wide array of pathogens and parasites. The MHC also plays a part in sexual mate selection, promoting genetic diversity in subsequent generations [72]. MHC class I loci present antigens from intracellular pathogens (such as viruses) while MHC II binds to those from extracellular parasites, although certain studies have sought to compare MHC II genetic constitution to virus occurrence in rodents [71, 73].

MHC typing of key *Mastomys* populations across West Africa could be carried out together with concurrent neutral microsatellite characterisation to tease apart patterns caused by genetic drift from those shaped by pathogen-mediated selection. This would improve our understanding relevant to topics such as whether there are specific MHC alleles or MHC supertypes (i.e., alleles with similar characteristics in the functional important antigen-binding sites) associated with LASV infection status [74]. For instance, several MHC II DQA alleles were detected to be likely involved in the susceptibility or the resistance of bank vole rodents *Myodes glareolus* to Puumala and Cowpox viral infections [75]. In fact, genotyping of MHC II DQB exon 2 in *M. natalensis* revealed 21 different alleles with strong signals of balancing selection on the peptide-binding sites, showing that DQB seems to be a good marker to investigate pathogen-driven selection [76].

Another point of interest would be to find out whether there is trans-species polymorphism between *M. natalensis* and *M. erythroleucus* concerning alleles connected to LASV infection status. Phylogenetic analysis of MHC II DRB variability in two murid rodents which are sympatric in European forests, the Wood mouse *Apodemus sylvaticus* and the Yellow-necked mouse *Apodemus flavicollis*, showed that the sequences did not separate according to species, consistent with trans-species evolution of the MHC in these taxa [77].

A broad overview of LASV lineage patterns in rodents across the West African region now allows us to conduct more intricate investigations on spatio-temporal strain circulation within specific hotspots, and to more closely compare virus sequences between rodents and humans. Accomplishing this will involve the continuation of intensive, longitudinal rodent sampling in localities where this has already begun, supplemented by ad hoc trapping to fill in the gaps where connectivity to human data is required. When possible, whole-genome sequencing of the LASV to enhance phylogenetic rigour should be effected. Furthermore, beyond mitochondrial DNA to designate rodent species, additional markers should be typed; such as neutral microsatellites for population structure and MHC to throw light on susceptibility to LASV.

## Lassa fever burden in West Africa

A study conducted in Sierra Leone and published in 1987 evaluated the LASV serology in humans and rodents. The study estimated that annually, 100,000–300,000 people are infected with LASV with around 5000 deaths in West Africa [53]. However, in the last 35 years, the population of Sub-Saharan Africa (SSA) has doubled [78] and crop production has intensified in the entire region resulting in severe damage to forests and ecosystems [79]. Updated models of LASV infection have estimated an annual incidence of 897,700 across West Africa with Nigeria, Ghana and Ivory Coast having the greatest number of infections and Sierra Leone, Nigeria and Guinea having the greatest rate per 100 people [80]. Although is still unclear how this anthropic action will impact on LF ecology, the estimated burden of LF is currently being investigated through a grant awarded in 2019 by the Coalition for Epidemic Preparedness Innovations [81].

Approximately 80% of LF infections are asymptomatic, while symptomatic cases present with a variety of symptoms ranging from milder flu-like symptoms, fever, muscle aches, sore throat, nausea, vomiting, chest- and abdominal pain, to severe haemorrhagic manifestations of the liver, spleen or kidneys which often result in death [8, 82]. The survivors of LF also have a high risk of developing neurological sequelae such as encephalopathy, paraparesis and, in approximately one-third of the cases, sensorineural hearing loss [83–87].

**Fig. 3:**
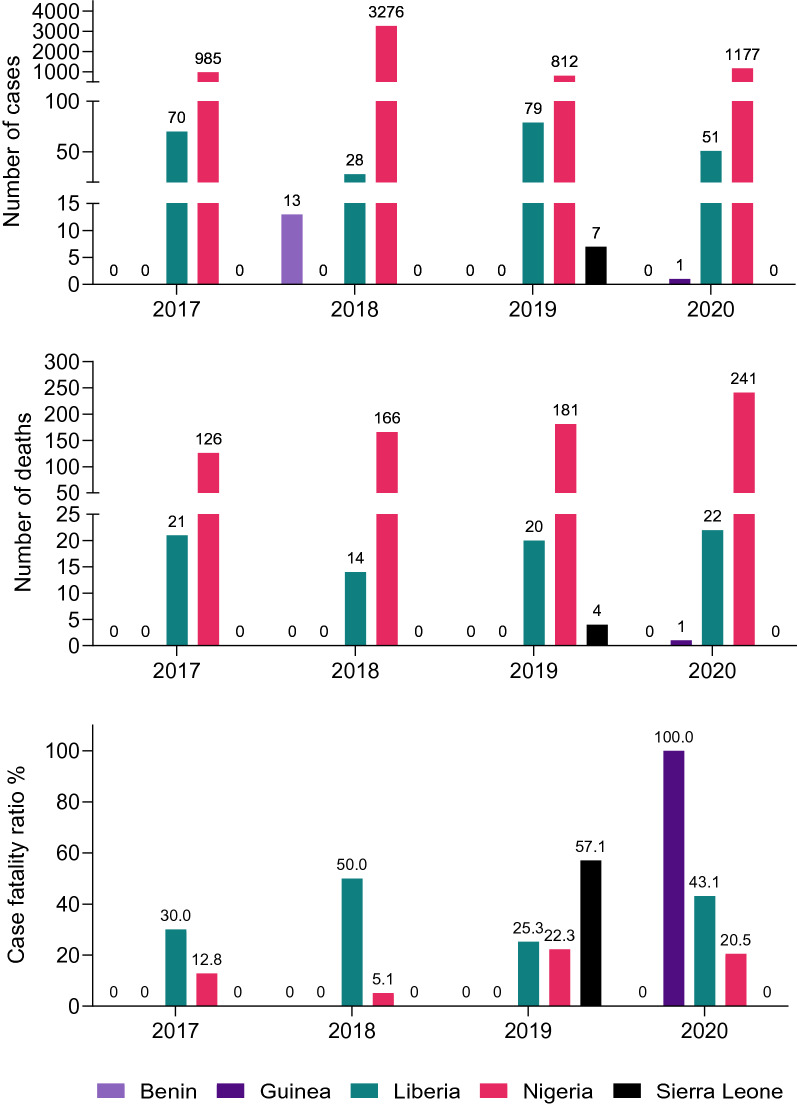
Lassa fever outbreaks from 2017 to 2020 eported by the WHO Regional Office for Africa**.** Nigeria and Liberia have had ongoing LF outbreaks since 2017. Although Nigeria reported a significantly higher number of cases, Liberia presented a proportionally higher number of deaths. High case fatality ratios for Sierra Leone and Guinea might be biased due to the low number of cases reported

Studies have suggested that LF outbreaks are likely due to independent zoonotic transmission events from infected rodent hosts, whilst approximately 20% of cases result from secondary human-to-human transmission especially through nosocomial transmission in hospital settings [15, 88]. Figure 3 depicts recent outbreaks reported by the WHO Regional Office for Africa, showing that Nigeria and Liberia have had ongoing LF outbreaks since 2017 [89–92]. Although Nigeria presents the largest burden in the number of cases, Liberia shows a higher case fatality ratio. This is potentially due to the efforts of the NCDC in improving LF diagnostics, allowing early treatment and healthcare management [11], in addition to many cases from Liberia and other countries not being detected or reported. For instance, the Program for Monitoring Emerging Diseases (ProMED, supportedby the International Society for Infectious Diseases (ISID) identified LF cases in Togo and Burkina Faso that were not included in WHO Afro reports [93].

Hence, the number of LF cases is challenging to accurately determine, as diagnostics tests are crucial for differential diagnoses [37, 88]. Asymptomatic or mild LF cases usually remain undiagnosed or misdiagnosed and unreported. These individuals may show symptoms that are similar to other endemic diseases and often do not seek formal medical attention. Furthermore, from the symptomatic cases who seek health care services only 20% may be captured by the surveillance systems. A fraction of the cases is missed due to atypical clinical presentation of the disease and lack of diagnostic capacity [19, 88, 94, 95]. LF is responsible for up to 10–16% of annual hospital admissions in some regions of Sierra Leone and Liberia [88, 96]. In Nigeria, a study evaluating admission and deaths among adults in a tertiary health facility and treatment centre showed that the proportion of admissions associated with LF increased ten-fold (0.3–3.4%) between 2001 and 2018 [37].

West Africa peri-urban areas have been witnessing an annual increase in the number of LF cases [19, 25, 37, 97–99]. A review of 102 outbreaks of the human disease reported a median seroprevalence of 13% in the communities studied. The study also reported a heterogenous seroprevalence despite the magnitude of the infection rate, including countries with fewer reported cases of the disease [100]. Systematic sampling to detect seroprevalence across the endemic region is currently planned but not yet completed [81]. A retrospective study performed on 675 samples obtained from suspected LF patients at the Kenema GovernmentHospital Lassa Diagnostic Laboratory (Sierra Leone) between 2007 and 2014 identified a seroprevalence of 50.2% [101]. A study carried out in Mali, following the first diagnosis of LF in a foreign national in 2009, provided evidence of ongoing transmission, where anti- LASV IgG seroprevalence ranged from 14.5 to 44% in three studied communities, even though no previous case of LF has been reported [102, 103]. In Liberia, the prevalence of antibody against LASV has been found to range from 15 to 20% [25].

The impact of LF in West Africa is believed to be underestimated and compounded by a paucity of data in many countries [37]. The case fatality ratio (CFR) from recent outbreaks in Africa, showed a mean CFR of 15% (Fig. 3), similar to the overall CFR of 1–15% shown in available epidemiological studies [10, 98, 104]. Although CFR tends to be higher among hospitalised patients, a study from a Nigerian hospital between 2001 and 2018 has shown a decline in the annual CFR from 94 to 15% [37].

Human-to-human transmission is most pronounced in healthcare settings, resulting in healthcare workers (HCW) having a high risk of infection and mortality [25, 29, 105, 106]. In the Nigerian LF outbreak in 2018, this occupational group represented 8% of all confirmed cases [25, 96, 105]. Atypical clinical presentation, low-suspicion of viral haemorrhagic fevers among those presenting to hospital, limited experience of LF specifically, inadequate availability of personal protective equipment and poor infection prevention and control practices are some of the factors associated with HCW infection. Consequently, HCWs caring for individual LF patients may reduce direct contact with patients, compromising these patients’ management and influencing disease-associated morbidity and mortality [106, 107].

A common complication/sequelae of LF is sensorineural hearing loss (SNHL), although its the prevalence and impact in endemic regions may be underestimated. Studies have shown that approximately one-third of survivors suffer from SNHL [37, 86]. Hearing loss has a socio-economic impact on survivors and public health, especially in endemic countries with limited capacity and resources for the support of individuals with sensory difficulties.

Treatment costs for LF infection may stretch the limited financial resources of patients who typically come from lower socio-economic positions within the community in endemic regions [11]. Prevention and control of LF would therefore significantly reduce the burden placed on those most affected. The public response to LF outbreaks is often a challenge to local authorities and national and international support is often required during a large outbreak [98, 108]. Thus, LF remains a public health challenge requiring continuous management effort by all stakeholders.

## Conclusions

LF infection may be considered a neglected tropical disease due to its localised occurrence in West Africa. However, its morbidity and mortality in the region represent a significant socio-economic and global health concern. In 2018, LF was added to the “WHO Research and Development Blueprint” portfolio, a global initiative for preparedness and rapid research on severe emerging diseases. The strategy promotes research and development on laboratory diagnostic tests, vaccines and treatment for epidemic threats [109]. A recent review reported the LASV vaccines under development, of which over 20 candidates were under preclinical phase in 2019 [110]. On this direction, Coalition for Epidemic Preparedness Innovations (CEPI) is currently promoting the development of six LASV vaccine candidates, two of which have entered phase I clinical trials: Themis Bioscience is testing a measles vector (MV) vaccine platform, while Inovio Pharmaceuticals is evaluating a DNA vaccine [111].

While a LASV vaccine is still unavailable, increasing insight into LF ecology, particularly where LASV rodent hosts are concerned, can provide a more dependable assessment of zoonotic risk. In this regard molecular analysis as a tool has proven useful, driving a series of advances in the mapping of LASV occurrence, lineage clustering and more accurate identification of rodent hosts [35]. Most of the findings so far have enabled virus and rodent genetic categorisation across the West African region at the ‘lineage’ level. As the overall quantity of molecular sequences grows, this review addresses avenues for future research connected with characterisation at a finer scale. These lines of investigation are directed at determining rodent genotypes especially susceptible to LASV infection and delineating virus strain assortments across space and time to improve tracking of rodent-human transmission and human-to-rodent transmission [35].

Furthermore, this review demonstrated that it is vital to incorporate system dynamics components in a broader One Health approach in order to identify appropriate interventions to control the critical driver of LF outbreaks. The integration of interdisciplinary methodologies including virologists, clinicians, veterinarians, epidemiologists, ecologists among others has shown how interconnected are human actions, climate and reservoir behaviour and how each of them affects the emergence and persistence of the disease in West Africa. Such approaches have also provided evidence and strategies that may be applied to restrain other zoonotic infections. Although humans are unable to control natural events associated with the (re)emergence of infectious diseases, embracing the responsibility of the anthropic impact will support well-elaborated and sustainable actions directed to prevent and control EID outbreak events.

## Data Availability

Not applicable.

## Abbreviations

CEPI: Coalition for Epidemic Preparedness Innovations
CFR: Case fatality ratio
COVID-19: Coronavirus disease 2019
EID: Emerging infectious disease(s)
HCW: Health care worker(s)
LASV: Lassa virus (*Lassa mammarenavirus*)
LF: Lassa fever
MHC: Major histocompatibility complex
NCDC: Nigeria Centre for Diseases Control
SNHL: Sensorineural hearing loss
SSA: Sub-Saharian Africa
WHO: World Health Organization

## References

[1] Kock RA (2013). Will the damage be done before we feel the heat? Infectious disease emergence and human response. Anim Health Res Rev.

[2] Karesh WB, Cook RA (2009). One world–one health. Clin Med (Lond).

[3] Kock R (2014). Drivers of disease emergence and spread: is wildlife to blame?. Onderstepoort J Vet Res.

[4] Bonwitt J, Kelly AH, Ansumana R, Agbla S, Sahr F, Saez AM, Borchert M, Kock R, Fichet-Calvet E (2016). Rat-atouille: a mixed method study to characterize rodent hunting and consumption in the context of Lassa fever. EcoHealth.

[5] Fichet-Calvet E, Johnson N (2014). Chapter 5—Lassa fever: a rodent-human interaction. The role of animals in emerging viral diseases.

[6] Ter Meulen J, Lukashevich I, Sidibe K, Inapogui A, Marx M, Dorlemann A, Yansane ML, Koulemou K, Chang-Claude J, Schmitz H (1996). Hunting of peridomestic rodents and consumption of their meat as possible risk factors for rodent-to-human transmission of Lassa virus in the Republic of Guinea. Am J Trop Med Hyg.

[7] Gunther S, Emmerich P, Laue T, Kuhle O, Asper M, Jung A, Grewing T, ter Meulen J, Schmitz H (2000). Imported lassa fever in Germany: molecular characterization of a new lassa virus strain. Emerg Infect Dis.

[8] Gunther S, Lenz O (2004). Lassa virus. Crit Rev Clin Lab Sci.

[9] WHO. Lassa fever disease outbreaks; 2020. http://www.who.int/csr/don/archive/disease/lassa_fever/en/. Accessed 30 Oct 2020.

[10] NCDC. Lassa fever outbreaks; 2020. https://ncdc.gov.ng/diseases/factsheet/47. Accessed 30 Oct 2020.

[11] Naidoo D, Ihekweazu C (2020). Nigeria's efforts to strengthen laboratory diagnostics—Why access to reliable and affordable diagnostics is key to building resilient laboratory systems. Afr J Lab Med.

[12] Bausch DG, Demby AH, Coulibaly M, Kanu J, Goba A, Bah A, Conde N, Wurtzel HL, Cavallaro KF, Lloyd E (2001). Lassa fever in Guinea: I. Epidemiology of human disease and clinical observations. Vector Borne Zoonotic Dis.

[13] Frame JD, Yalley-Ogunro JE, Hanson AP (1984). Endemic Lassa fever in Liberia. V. Distribution of Lassa virus activity in Liberia: hospital staff surveys. Trans R Soc Trop Med Hyg.

[14] Kamara FK, Lahai RC, Babawo L, Kangbai JB (2020). Lassa fever in post-Ebola Sierra Leone: sociodemographics and case fatality rates of in-hospital patients admitted at the Kenema Government Hospital Lassa Fever Ward between 2016–2018. J Virol Pathog.

[15] Ogbu O, Ajuluchukwu E, Uneke CJ (2007). Lassa fever in West African sub-region: an overview. J Vector Borne Dis.

[16] Andersen KG, Shapiro BJ, Matranga CB, Sealfon R, Lin AE, Moses LM, Folarin OA, Goba A, Odia I, Ehiane PE (2015). Clinical sequencing uncovers origins and evolution of Lassa virus. Cell.

[17] Ehichioya DU, Hass M, Becker-Ziaja B, Ehimuan J, Asogun DA, Fichet-Calvet E, Kleinsteuber K, Lelke M, ter Meulen J, Akpede GO (2011). Current molecular epidemiology of Lassa virus in Nigeria. J Clin Microbiol.

[18] Fichet-Calvet E, Rogers DJ (2009). Risk maps of Lassa fever in West Africa. PLoS Negl Trop Dis.

[19] Mylne AQ, Pigott DM, Longbottom J, Shearer F, Duda KA, Messina JP, Weiss DJ, Moyes CL, Golding N, Hay SI (2015). Mapping the zoonotic niche of Lassa fever in Africa. Trans R Soc Trop Med Hyg.

[20] Ibukun FI (2020). Inter-lineage variation of Lassa virus glycoprotein epitopes: a challenge to Lassa virus vaccine development. Viruses.

[21] Kofman A, Choi MJ, Rollin PE (2019). Lassa fever in travelers from West Africa, 1969–2016. Emerg Infect Dis.

[22] Mazzola LT, Kelly-Cirino C (2019). Diagnostics for Lassa fever virus: a genetically diverse pathogen found in low-resource settings. BMJ Glob Health.

[23] Monadjem A, Taylor PJ, Denys C, Cotterill FPD: Rodents of sub-saharan Africa: De Gruyter; 2015.

[24] Shaffer JG, Grant DS, Schieffelin JS, Boisen ML, Goba A, Hartnett JN, Levy DC, Yenni RE, Moses LM, Fullah M (2014). Lassa fever in post-conflict sierra leone. PLoS Negl Trop Dis.

[25] Wiley MR, Fakoli L, Letizia AG, Welch SR, Ladner JT, Prieto K, Reyes D, Espy N, Chitty JA, Pratt CB (2019). Lassa virus circulating in Liberia: a retrospective genomic characterisation. Lancet Infect Dis.

[26] Overbosch F, de Boer M, Veldkamp KE, Ellerbroek P, Bleeker-Rovers CP, Goorhuis B, van Vugt M, van der Eijk A, Leenstra T, Khargi M (2020). Public health response to two imported, epidemiologically related cases of Lassa fever in the Netherlands (ex Sierra Leone), November 2019. Euro Surveill.

[27] McElroy AK, Akondy RS, Harmon JR, Ellebedy AH, Cannon D, Klena JD, Sidney J, Sette A, Mehta AK, Kraft CS (2017). A case of human lassa virus infection with robust acute T-cell activation and long-term virus-specific T-cell responses. J Infect Dis.

[28] Quigley RL: How, where and why do we evacuate those infected with viral hemorrhagic fevers? (110). Am J Trop Med Hygiene 2010; 83: 1–69. vol. 5_Suppl].

[29] ECDC. Rapid risk assessment: Cases of Lassa fever in the Netherlands ex Sierra Leone; 2019. https://www.ecdc.europa.eu/en/publications-data/rapid-risk-assessment-cases-lassa-fever-netherlands-ex-sierra-leone. Accessed 30 Oct 2020.

[30] Tuite AR, Watts AG, Kraemer MUG, Khan K, Bogoch II (2019). Potential for seasonal Lassa fever case exportation from Nigeria. Am J Trop Med Hyg.

[31] Fichet-Calvet E, Olschlager S, Strecker T, Koivogui L, Becker-Ziaja B, Camara AB, Soropogui B, Magassouba N, Gunther S (2016). Spatial and temporal evolution of Lassa virus in the natural host population in Upper Guinea. Sci Rep.

[32] Marien J, Lo Iacono G, Rieger T, Magassouba N, Gunther S, Fichet-Calvet E (2020). Households as hotspots of Lassa fever? Assessing the spatial distribution of Lassa virus-infected rodents in rural villages of Guinea. Emerg Microbes Infect.

[33] Kafetzopoulou LE, Pullan ST, Lemey P, Suchard MA, Ehichioya DU, Pahlmann M, Thielebein A, Hinzmann J, Oestereich L, Wozniak DM (2019). Metagenomic sequencing at the epicenter of the Nigeria 2018 Lassa fever outbreak. Science.

[34] Siddle KJ, Eromon P, Barnes KG, Mehta S, Oguzie JU, Odia I, Schaffner SF, Winnicki SM, Shah RR, Qu J (2018). Genomic analysis of Lassa virus during an increase in cases in Nigeria in 2018. N Engl J Med.

[35] Olayemi A, Adesina AS, Strecker T, Magassouba N, Fichet-Calvet E (2020). Determining ancestry between rodent- and human-derived virus sequences in endemic foci: towards a more integral molecular epidemiology of Lassa fever within West Africa. Biology (Basel).

[36] Monath TP (1975). Lassa fever: review of epidemiology and epizootiology. Bull World Health Organ.

[37] Akpede GO, Asogun DA, Okogbenin SA, Dawodu SO, Momoh MO, Dongo AE, Ike C, Tobin E, Akpede N, Ogbaini-Emovon E (2019). Caseload and case fatality of Lassa fever in Nigeria, 2001–2018: a specialist center's experience and its implications. Front Public Health.

[38] Asogun DA, Adomeh DI, Ehimuan J, Odia I, Hass M, Gabriel M, Olschlager S, Becker-Ziaja B, Folarin O, Phelan E (2012). Molecular diagnostics for lassa fever at Irrua specialist teaching hospital, Nigeria: lessons learnt from two years of laboratory operation. PLoS Negl Trop Dis.

[39] Fichet-Calvet E, Lecompte E, Koivogui L, Soropogui B, Dore A, Kourouma F, Sylla O, Daffis S, Koulemou K, Ter Meulen J (2007). Fluctuation of abundance and Lassa virus prevalence in Mastomys natalensis in Guinea West Africa. Vector Borne Zoonotic Dis.

[40] Keane E, Gilles HM (1977). Lassa fever in Panguma Hospital, Sierra Leone, 1973–6. Br Med J.

[41] Fichet-Calvet E, Lecompte E, Koivogui L, Daffis S, ter Meulen J (2008). Reproductive characteristics of Mastomys natalensis and Lassa virus prevalence in Guinea, West Africa. Vector Borne Zoonotic Dis.

[42] Akhmetzhanov AR, Asai Y, Nishiura H (2019). Quantifying the seasonal drivers of transmission for Lassa fever in Nigeria. Philos Trans R Soc Lond B Biol Sci.

[43] Marien J, Kourouma F, Magassouba N, Leirs H, Fichet-Calvet E (2018). Movement patterns of small rodents in Lassa fever-endemic villages in Guinea. EcoHealth.

[44] Olayemi A, Obadare A, Oyeyiola A, Fasogbon S, Igbokwe J, Igbahenah F, Ortsega D, Günther S, Verheyen E, Fichet-Calvet E (2018). Small mammal diversity and dynamics within Nigeria, with emphasis on reservoirs of the lassa virus. Syst Biodivers.

[45] Muehlenbein MP (2016). Disease and human/animal interactions. Annu Rev Anthropol.

[46] Bonwitt J, Saez AM, Lamin J, Ansumana R, Dawson M, Buanie J, Lamin J, Sondufu D, Borchert M, Sahr F (2017). At home with mastomys and rattus: human-rodent interactions and potential for primary transmission of Lassa virus in domestic spaces. Am J Trop Med Hyg.

[47] Tambo E, Adetunde OT, Olalubi OA (2018). Re-emerging Lassa fever outbreaks in Nigeria: Re-enforcing "One Health" community surveillance and emergency response practice. Infect Dis Poverty.

[48] United Nations. World population prospects - Population Division; 2019. https://population.un.org/wpp/. Accessed 30 Oct 2020.

[49] Hitimana J, Kiyiapi JLO, Kibugi PW, Kisioh H, Mayienda R, Warinwa F, Lenaiyasa P, Sumba D, Grillo O, Venora G (2011). Challenges of linking socio-economic significance and conservation value of forests in drylands of Kenya: case study of kirisia forest-samburu pastoralists coexistence. Biological diversity and sustainable resources use.

[50] Sluydts V, Crespin L, Davis S, Lima M, Leirs H (2007). Survival and maturation rates of the African rodent, Mastomys natalensis: density-dependence and rainfall. Integr Zool.

[51] Cook KH, Vizy EK (2019). Contemporary climate change of the african monsoon systems. Curr Clim Change Rep.

[52] Monath TP, Newhouse VF, Kemp GE, Setzer HW, Cacciapuoti A (1974). Lassa virus isolation from Mastomys natalensis rodents during an epidemic in Sierra Leone. Science.

[53] McCormick JB, Webb PA, Krebs JW, Johnson KM, Smith ES (1987). A prospective study of the epidemiology and ecology of Lassa fever. J Infect Dis.

[54] Walker DH, Wulff H, Lange JV, Murphy FA (1975). Comparative pathology of Lassa virus infection in monkeys, guinea-pigs, and Mastomys natalensis. Bull World Health Organ.

[55] Fiedler LA (1988). Rodent pest problems and management in Eastern Africa. Bulletin Phytosanitaire de la FAO.

[56] Granjon LMndhn, Paris (France). Lab. de zoologie, mammiferes et oiseaux), Duplantier JM, Catalan J, Britton-Davidian J: Systematics of the genus Mastomys (Thomas, 1915) (Rodentia: Muridae). A review. oct1997, v. 127.

[57] Green CA, Keogh H, Gordon DH, Pinto M, Hartwig EK (1980). The distribution, identification, and naming of the Mastomys natalensis species complex in southern Africa (Rodentia: Muridae). J Zool.

[58] Leirs H, développement BAgdlca, Project T-BJRR: Population Ecology of Mastomys Natalensis (Smith, 1834): Implications for Rodent Control in Africa: Belgian Administration for Development Cooperation; 1994.

[59] Coetzee CG (1975). The biology, behaviour, and ecology of Mastomys natalensis in southern Africa. Bull World Health Organ.

[60] Happold D, Hoffmann M, Butynski T, Kingdon J, Happold D (2013). Mammals of Africa. Mammals of Africa rodents hares and rabbits.

[61] Kronmann KC, Nimo-Paintsil S, Guirguis F, Kronmann LC, Bonney K, Obiri-Danso K, Ampofo W, Fichet-Calvet E (2013). Two novel arenaviruses detected in pygmy mice, Ghana. Emerg Infect Dis.

[62] Olayemi A, Cadar D, Magassouba N, Obadare A, Kourouma F, Oyeyiola A, Fasogbon S, Igbokwe J, Rieger T, Bockholt S (2016). New hosts of the Lassa virus. Sci Rep.

[63] Yadouleton A, Agolinou A, Kourouma F, Saizonou R, Pahlmann M, Bedie SK, Bankole H, Becker-Ziaja B, Gbaguidi F, Thielebein A (2019). Lassa virus in Pygmy Mice, Benin, 2016–2017. Emerg Infect Dis.

[64] Wulff H, Fabiyi A, Monath TP (1975). Recent isolations of Lassa virus from Nigerian rodents. Bull World Health Organ.

[65] Fichet-Calvet E, Becker-Ziaja B, Koivogui L, Gunther S (2014). Lassa serology in natural populations of rodents and horizontal transmission. Vector Borne Zoonotic Dis.

[66] Olayemi A, Oyeyiola A, Obadare A, Igbokwe J, Adesina AS, Onwe F, Ukwaja KN, Ajayi NA, Rieger T, Gunther S (2018). Widespread arenavirus occurrence and seroprevalence in small mammals, Nigeria. Parasit Vectors.

[67] Olayemi A, Fichet-Calvet E (2020). Systematics, ecology, and host switching: attributes affecting emergence of the Lassa virus in rodents across Western Africa. Viruses.

[68] Nicolas V, Schaeffer B, Missoup AD, Kennis J, Colyn M, Denys C, Tatard C, Cruaud C, Laredo C (2012). Assessment of three mitochondrial genes (16S, Cytb, CO1) for identifying species in the Praomyini tribe (Rodentia: Muridae). PLoS ONE.

[69] Olayemi A, Obadare A, Oyeyiola A, Igbokwe J, Fasogbon A, Igbahenah F, Ortsega D, Asogun D, Umeh P, Vakkai I (2016). Arenavirus diversity and phylogeography of mastomys natalensis rodents, Nigeria. Emerg Infect Dis.

[70] Lalis A, Leblois R, Lecompte E, Denys C, Ter Meulen J, Wirth T (2012). The impact of human conflict on the genetics of Mastomys natalensis and Lassa virus in West Africa. PLoS ONE.

[71] Sommer S (2005). The importance of immune gene variability (MHC) in evolutionary ecology and conservation. Front Zool.

[72] Santos PS, Courtiol A, Heidel AJ, Honer OP, Heckmann I, Nagy M, Mayer F, Platzer M, Voigt CC, Sommer S (2016). MHC-dependent mate choice is linked to a trace-amine-associated receptor gene in a mammal. Sci Rep.

[73] Piertney SB, Oliver MK (2006). The evolutionary ecology of the major histocompatibility complex. Heredity (Edinb).

[74] Qurkhuli T, Schwensow N, Brandel SD, Tschapka M, Sommer S (2019). Can extreme MHC class I diversity be a feature of a wide geographic range? The example of Seba's short-tailed bat (Carollia perspicillata). Immunogenetics.

[75] Deter J, Bryja J, Chaval Y, Galan M, Henttonen H, Laakkonen J, Voutilainen L, Vapalahti O, Vaheri A, Salvador AR (2008). Association between the DQA MHC class II gene and Puumala virus infection in Myodes glareolus, the bank vole. Infect Genet Evol.

[76] GouydeBellocq J, Leirs H (2010). Polymorphism and signatures of selection in the multimammate rat DQB gene. Immunogenetics.

[77] Musolf K, Meyer-Lucht Y, Sommer S (2004). Evolution of MHC-DRB class II polymorphism in the genus Apodemus and a comparison of DRB sequences within the family Muridae (Mammalia: Rodentia). Immunogenetics.

[78] The World Bank. Sub-Saharan population; 2020. https://data.worldbank.org/indicator/SP.POP.TOTL?locations=ZG. Accessed 30 Oct 2020.

[79] van Loon MP, Hijbeek R, Ten Berge HFM, De Sy V, Ten Broeke GA, Solomon D, van Ittersum MK (2019). Impacts of intensifying or expanding cereal cropping in sub-Saharan Africa on greenhouse gas emissions and food security. Glob Chang Biol.

[80] Basinski AJ, Fichet-Calvet E, Sjodin AR, Varrelman TJ, Remien CH, Layman NC (2021). Bridging the gap: Using reservoir ecology and human serosurveys to estimate Lassa virus spillover in West Africa. PLoS Comput Biol.

[81] CEPI. Lassa epidemiological studies for preparation of clinical trials in affected countries - Expression of Interest; 2018. https://cepi.net/wp-content/uploads/2019/02/180701-Expression-of-Interest-Lassa_FINAL-1.pdf. Accessed 30 Oct 2020.

[82] Oti VB, Rodriguez-Morales AJ (2018). A reemerging Lassa virus: aspects of its structure, replication, pathogenicity and diagnosis. Current topics in tropical emerging diseases and travel medicine.

[83] Duvignaud A, Doutchi M, Abejegah C, Etafo I, Jaspard M, Serra B, Tricaud E, Levy-Marchal C, Anglaret X, Ahmed LA (2020). Delayed-onset paraparesis in Lassa fever: a case report. Int J Infect Dis.

[84] Gunther S, Weisner B, Roth A, Grewing T, Asper M, Drosten C, Emmerich P, Petersen J, Wilczek M, Schmitz H (2001). Lassa fever encephalopathy: Lassa virus in cerebrospinal fluid but not in serum. J Infect Dis.

[85] Hallam SJ, Koma T, Maruyama J, Paessler S (2018). Review of mammarenavirus biology and replication. Front Microbiol.

[86] Mateer EJ, Huang C, Shehu NY, Paessler S (2018). Lassa fever-induced sensorineural hearing loss: a neglected public health and social burden. PLoS Negl Trop Dis.

[87] Paessler S, Walker DH (2013). Pathogenesis of the viral hemorrhagic fevers. Annu Rev Pathol.

[88] WHO Africa. Lassa fever fact sheet; 2018. https://www.who.int/news-room/fact-sheets/detail/lassa-fever. Accessed 30 Oct 2020.

[89] WHO Africa. Weekly bulletin on outbreaks and other emergencies Week 52 2017; 2017. http://apps.who.int/iris/bitstream/handle/10665/259794/OEW52-2329122017.pdf?sequence=1. Accessed 30 Oct 2020

[90] WHO Africa. Weekly bulletin on outbreaks and other emergencies Week 52 2018; 2018. http://apps.who.int/iris/bitstream/handle/10665/277423/OEW52-2228122018.pdf. Accessed 30 Oct 2020.

[91] WHO Africa. Weekly bulletin on outbreaks and other emergencies Week 52 2019; 2019. https://apps.who.int/iris/bitstream/handle/10665/330351/OEW52-31122019.pdf. Accessed 30 Oct 2020.

[92] WHO Africa. Weekly bulletin on outbreaks and other emergencies Week 51 2020; 2020. https://apps.who.int/iris/bitstream/handle/10665/338051/OEW51-1420122020.pdf. Accessed 18 Feb 2021.

[93] ProMED. Program for monitoring emerging diseases; 2021. https://promedmail.org/. Accessed 18 Feb 2021.

[94] Akhuemokhan OC, Ewah-Odiase RO, Akpede N, Ehimuan J, Adomeh DI, Odia I, Olomu SC, Pahlmann M, Becker-Ziaja B, Happi CT (2017). Prevalence of Lassa Virus Disease (LVD) in Nigerian children with fever or fever and convulsions in an endemic area. PLoS Negl Trop Dis.

[95] Burki T (2018). Lassa fever in Nigeria: the great unknown. Lancet.

[96] Woyessa AB, Maximore L, Keller D, Dogba J, Pajibo M, Johnson K, Saydee E, Monday J, Tuopileyi R, Mahmoud N (2019). Lesson learned from the investigation and response of Lassa fever outbreak, Margibi County, Liberia, 2018: case report. BMC Infect Dis.

[97] ACDC. Lassa fever; 2020. https://africacdc.org/disease/lassa-fever/. Accessed 30 Oct 2020.

[98] Ilori EA, Frank C, Dan-Nwafor CC, Ipadeola O, Krings A, Ukponu W, Womi-Eteng OE, Adeyemo A, Mutbam SK, Musa EO (2019). Increase in Lassa fever cases in Nigeria, January–March 2018. Emerg Infect Dis.

[99] NCDC. Lassa fever situation report epidemiological week 52; 2020. https://ncdc.gov.ng/themes/common/files/sitreps/e84fd2d2febe5ccd892420e1d92f630a.pdf. Accessed 30 Oct 2020.

[100] Gibb R, Moses LM, Redding DW, Jones KE (2017). Understanding the cryptic nature of Lassa fever in West Africa. Pathog Glob Health.

[101] O'Hearn AE, Voorhees MA, Fetterer DP, Wauquier N, Coomber MR, Bangura J, Fair JN, Gonzalez JP, Schoepp RJ (2016). Serosurveillance of viral pathogens circulating in West Africa. Virol J.

[102] Baumann J, Knupfer M, Ouedraogo J, Traore BY, Heitzer A, Kane B, Maiga B, Sylla M, Kouriba B, Wolfel R (2019). Lassa and crimean-congo hemorrhagic fever viruses, Mali. Emerg Infect Dis.

[103] Sogoba N, Rosenke K, Adjemian J, Diawara SI, Maiga O, Keita M, Konate D, Keita AS, Sissoko I, Boisen M (2016). Lassa virus seroprevalence in Sibirilia Commune, Bougouni District, Southern Mali. Emerg Infect Dis.

[104] Ilori EA, Furuse Y, Ipadeola OB, Dan-Nwafor CC, Abubakar A, Womi-Eteng OE, Ogbaini-Emovon E, Okogbenin S, Unigwe U, Ogah E (2019). Epidemiologic and clinical features of Lassa fever outbreak in Nigeria, January 1–May 6, 2018. Emerg Infect Dis.

[105] Dan-Nwafor CC, Ipadeola O, Smout E, Ilori E, Adeyemo A, Umeokonkwo C, Nwidi D, Nwachukwu W, Ukponu W, Omabe E (2019). A cluster of nosocomial Lassa fever cases in a tertiary health facility in Nigeria: description and lessons learned, 2018. Int J Infect Dis.

[106] Fisher-Hoch SP, Tomori O, Nasidi A, Perez-Oronoz GI, Fakile Y, Hutwagner L, McCormick JB (1995). Review of cases of nosocomial Lassa fever in Nigeria: the high price of poor medical practice. BMJ.

[107] Ijarotimi IT, Ilesanmi OS, Aderinwale A, Abiodun-Adewusi O, Okon IM (2018). Knowledge of Lassa fever and use of infection prevention and control facilities among health care workers during Lassa fever outbreak in Ondo State, Nigeria. Pan Afr Med J.

[108] Dan-Nwafor CC, Furuse Y, Ilori EA, Ipadeola O, Akabike KO, Ahumibe A, Ukponu W, Bakare L, Okwor TJ, Joseph G (2019). Measures to control protracted large Lassa fever outbreak in Nigeria, 1 January to 28 April 2019. Euro Surveill.

[109] Mehand MS, Al-Shorbaji F, Millett P, Murgue B (2018). The WHO R&D Blueprint: 2018 review of emerging infectious diseases requiring urgent research and development efforts. Antiviral Res.

[110] Salami K, Gouglas D, Schmaljohn C, Saville M, Tornieporth N (2019). A review of Lassa fever vaccine candidates. Curr Opin Virol.

[111] CEPI. Priority diseases - Lassa; 2021. https://cepi.net/research_dev/priority-diseases/. Accessed 18 Feb 2021.

